# Learning Peri-saccadic Remapping of Receptive Field from Experience in Lateral Intraparietal Area

**DOI:** 10.3389/fncom.2017.00110

**Published:** 2017-11-28

**Authors:** Xiao Wang, Yan Wu, Mingsha Zhang, Si Wu

**Affiliations:** State Key Laboratory of Cognitive Neuroscience and Learning, IDG/McGovern Institute for Brain Research, Beijing Normal University, Beijing, China

**Keywords:** predictive remapping, saccade, STDP, LIP, corollary discharge

## Abstract

Our eyes move constantly at a frequency of 3–5 times per second. These movements, called saccades, induce the sweeping of visual images on the retina, yet we perceive the world as stable. It has been suggested that the brain achieves this visual stability via predictive remapping of neuronal receptive field (RF). A recent experimental study disclosed details of this remapping process in the lateral intraparietal area (LIP), that is, about the time of the saccade, the neuronal RF expands along the saccadic trajectory temporally, covering the current RF (CRF), the future RF (FRF), and the region the eye will sweep through during the saccade. A cortical wave (CW) model was also proposed, which attributes the RF remapping as a consequence of neural activity propagating in the cortex, triggered jointly by a visual stimulus and the corollary discharge (CD) signal responsible for the saccade. In this study, we investigate how this CW model is learned naturally from visual experiences at the development of the brain. We build a two-layer network, with one layer consisting of LIP neurons and the other superior colliculus (SC) neurons. Initially, neuronal connections are random and non-selective. A saccade will cause a static visual image to sweep through the retina passively, creating the effect of the visual stimulus moving in the opposite direction of the saccade. According to the spiking-time-dependent-plasticity rule, the connection path in the opposite direction of the saccade between LIP neurons and the connection path from SC to LIP are enhanced. Over many such visual experiences, the CW model is developed, which generates the peri-saccadic RF remapping in LIP as observed in the experiment.

## Introduction

Our eyes move constantly at a frequency of 3–5 times per second. An eye movement, called a saccade, induces the sweeping of visual images on the retina, yet we perceive the world to be stable. The shift of visual inputs on the retina caused by a saccade is no different to a shift caused by objects moving in real space, yet we do not mistake one for another. Understanding how the brain achieves visual stability across the saccade has been a challenge to both experimental (Sommer and Wurtz, 2008; Hall and Colby, 2011; Wurtz et al., 2011) and theoretical (Quaia et al., 1998; Keith et al., 2010; Ziesche and Hamker, 2014) neuroscience for decades. It has been suggested that the brain solves this problem by utilizing an efference copy of the motor command responsible for a saccade, called corollary discharge (CD), to compensate in advance for the disturbance brought by the saccade (Sommer and Wurtz, 2002, 2006; Sun and Goldberg, 2016). This idea is supported by a phenomenon found in the lateral intraparietal cortex (LIP) called peri-saccadic receptive field (RF) remapping, which shows that neurons can respond to stimuli appearing in their future receptive fields (FRFs), i.e., the spatial locations the neuronal RFs will move into after the saccade, even before the eye movement actually starts.

**Figure 1:**
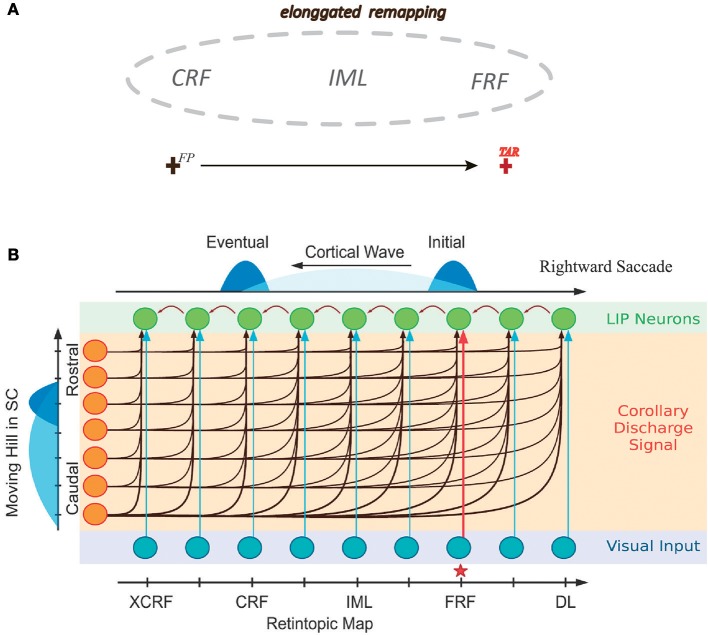
Illustrating elongated peri-saccadic remapping of neuronal RF in LIP and the CW model. **(A)** In the peri-saccadic period, neuronal RF in LIP transiently “stretches” along the saccadic trajectory, covering CRF, FRF, and the intermediate location (IML) between them. **(B)** Cartoon of the one-dimensional CW model. Neurons in LIP are uni-directionally connected in the opposite direction of the saccade. Each neuron in LIP (green) receives an input from its adjacent neuron and the CD signal from the moving hill activity in SC (left). About the time of the saccade, the joint effort of the visual stimulus at FRF of an example neuron (red arrow from the input layer) and the CD signal triggers a wave of excitation to emerge and propagate along the opposite direction of the saccade and eventually reach to CRF of the neuron. Adapted from Wang et al. (2016).

**Figure 2:**
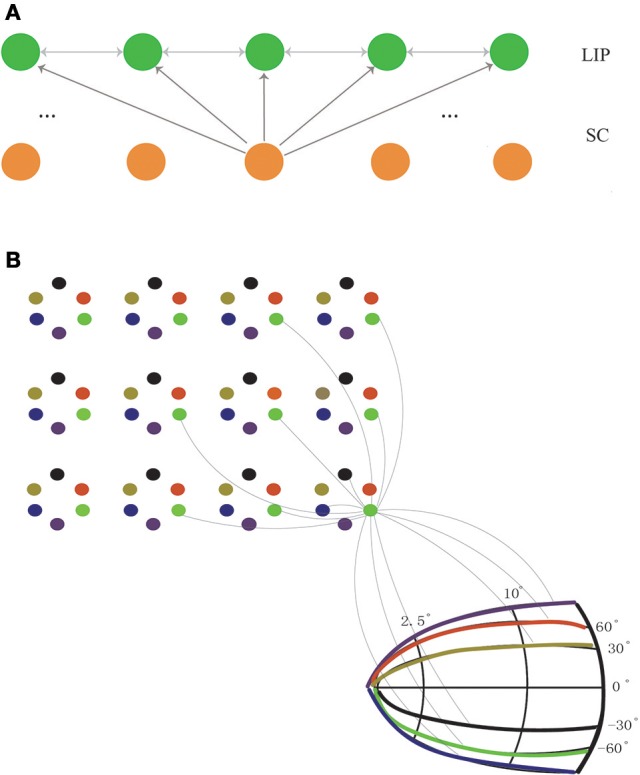
The network structure before learning. **(A)** The 1D network model. Neurons in LIP (only excitatory neurons are shown for clearance) are uniformly distributed on the 1D retinotopic map, and neurons in SC are on the 1D movement map. The connections between LIP neurons are bidirectional and weak (indicated by gray lines), and so are the feedforward connections from SC to LIP. **(B)** The 2D network model. Neurons in LIP are located on the 2D retinotopic map (grid). At each location, 6 excitatory neurons are considered. Their connections are bidirectional and weak. Neurons in SC are located on the 2D movement map (in the polar coordinate). The connections from SC to LIP are homogenous and non-selective. Since there are too many connections between neurons, for clearance, we only show the connections of an example neuron in LIP to other neurons in LIP and SC, and since the connections are random (see Equation 14), only a few of them are presented.

In a recent study Wang et al. (2016) further investigated the detailed time course of peri-saccadic remapping in LIP. They found that about the time of a saccade, the neuronal RF expands along the saccadic trajectory temporally, covering the current RF (CRF), the future RF (FRF), and the region the eye will sweep through during the saccade (Figure 1A). Moreover, the RF of the neuron shrinks to its normal size shortly after the saccade. Wang et al. further proposed a computational model to unveil the underlying mechanism of this remapping phenomenon. Their model attributes the peri-saccadic RF remapping to the neural activity propagating in LIP, triggered by visual stimuli and the CD signal (Figure 1B). Hereafter, the model is referred to as the cortical wave (CW) model for convenience. Specifically, the CW model assumes that: (1) neurons in the LIP are grouped into many clusters, and neurons in each cluster are uni-directionally connected to form a path responsible for RF remapping in the opposite direction (Figure 1B); (2) the CD signal from the superior colliculus (SC) conveys the information about the saccadic direction and amplitude; (3) the joint effect of a visual stimulus and the CD signal triggers the neural activity to propagate along the opposite direction of the saccade (i.e., CW), achieving peri-saccadic remapping. The CW model reproduces the experimental data and also outperforms other models with a simpler structure (Quaia et al., 1998).

Despite its success in interpreting the experimental data, the biological plausibility of the CW model remains unresolved. In particular, the model makes a few strong assumptions about the network topology, whose biological relevance, such as the uni-directional connections between neurons in LIP and the matched connections from SC to LIP, is yet to be justified. In this study, we aim to address this issue by computational modeling. Specifically, we build up a network-learning model to demonstrate that the structure of the CW model can be naturally acquired from visual experiences at the development of the brain via biologically plausible synaptic plasticity. We expect this study will strengthen our understanding on the occurrence of RF remapping.

## Materials and methods

### The network model

The network model we consider consists of two layers of neurons, one for LIP and one for SC. The final structure of the network will be learned from visual experiences via a biologically plausible synaptic plasticity rule (to be introduced below). Initially, the connections between LIP neurons and the connections from SC to LIP are homogenous and un-structured (Figure 2). For the purpose of elucidating the learning process clearly, we first study a simplified one-dimensional (1D) network model (Figure 2A), and later generalize the study to the 2D case (Figure 2B).

In the 1D network, neurons in the LIP layer are located in a 1D retinotopic map. They are uniformly distributed in the 1D retinotopic map, with inhibitory neurons inserted among excitatory ones at regular intervals. Before learning, neuronal connections between LIP neurons are bi-directional and stochastic. Neurons in the SC layer are uniformly distributed in a 1D movement map and send the CD signal containing the saccade information to LIP neurons. The connections from SC to LIP which are weak before learning will be strengthened after learning. Note that the connection selectivity from SC to LIP can only be reflected in the 2D network.

In the 2D network, neurons in LIP are distributed in the 2D retinotopic map. At each retinotopic location, rather than a single neuron, a group of remapping neurons exist (6 neurons are considered in the present study as an example). Before learning, similar to the 1D case, the connections between neurons at different spatial locations are bidirectional and random. After learning, neurons at the same location will be diversified to be responsible for different remapping directions. Neurons in the SC layer are located in the 2D movement map. Before learning, the connections from SC to LIP are weak and non-selective. After learning, the matched connections from SC to LIP are established.

### The synaptic plasticity

Two types of synaptic plasticity are considered in the present study. One is short-term plasticity (STP) (Markram and Tsodyks, 1996), which may display dominating short-term depression (STD), or dominating short-term facilitation (STF), or a mixture of both, depending on the parameters (Tsodyks et al., 1998). STP is not really essential for our model. We consider it because STP is observed ubiquitously in the experimental data (Stevens and Wang, 1995; Zucker and Regehr, 2002), and STD helps to stabilize the cortical wave. The detailed implementation of STP is presented in Equations (9–13).

We also consider long-term plasticity, which is key to learn the remapping function from visual experiences in our model. In particular, we consider spike-timing-dependent-plasticity (STDP), a biologically plausible learning rule (Markram et al., 1997; Bi and Poo, 1998; Tsodyks et al., 1998; Andrew, 2003; Dan and Poo, 2006) exclusive to synapses between excitatory neurons. Denote *W*_*ij*_(*t*)∈(0, 1) the synapse efficacy from neurons *i* to *j*. The STDP rule is given by

(1) $$\begin{array}{l} dW_{ij}(t)=\{\begin{array}{c} A_{+}e^{\frac{-\Delta t_{ij}}{\tau_{+}}},if\Delta t_{ij}\geq 0,\\ A_{-}e^{\frac{\Delta t_{ij}}{\tau_{-}}},if\Delta t_{ij}<0, \end{array} \end{array}$$

where *A*_+_ and *A*_−_ represent, respectively, the learning rates of facilitation and depression, and τ_+_ and τ_−_ the corresponding time constants. Δ*t*_*ij*_ denotes the time difference between the spiking moments of the postsynaptic neuron *i* and the presynaptic neuron *j*. Only nearest-neighbor spikes are counted in STDP (Izhikevich and Desai, 2003; Sjöström and Gerstner, 2010).

### The network dynamics

We adopt the AdEx IAF model with spike-frequency adaptation to describe the single neuron dynamics, which are given by Brette and Gerstner (2005)

(2) $$\begin{array}{l} C_{m}\frac{dV_{i}}{dt}=-g_{L}\left(V_{i}-E_{l}\right)+g_{L}\Delta_{T}\exp\left(\frac{V_{i}-V_{T}}{\Delta_{T}}\right)\\ +\;I_{i}^{rec}\left(t\right)+I_{i}^{ext}\left(t\right)-w_{i}, \end{array}$$

(3) $$\begin{array}{l} \tau_{w}\frac{dw_{i}}{dt}=a\left(V_{i}-E_{l}\right)-w_{i}, \end{array}$$

(4) $$\begin{array}{l} I_{i}^{ext}\left(t\right)=I_{i}^{bg}\left(t\right)+I_{i}^{sti}\left(t\right)+I_{i}^{SC}\left(t\right), \end{array}$$

(5) $$\begin{array}{l} I_{i}^{rec}\left(t\right)=I_{i}^{AMPA}\left(t\right)+I_{i}^{NMDA}\left(t\right)+I_{i}^{GABA}\left(t\right), \end{array}$$

where *V*_*i*_ is the membrane potential of neuron *i*, and *V*_*T*_ the firing threshold. When *V*_*i*_ > *V*_*T*_, the neuron fires and its membrane voltage is reset to be *V*_*re*_. *C*_*m*_ is the membrane capacitance, *g*_*L*_ the leak conductance, and *E*_*l*_ is the resting potential. *w*_*i*_ denotes the adaptation current, which increases following a spike. *I*^*rec*^ denotes the recurrent input from neurons in the LIP layer. *I*^*ext*^ denotes the external input, which consists of the background noise *I*^*bg*^, the visual stimulus *I*^*sti*^, and the CD signal *I*^*SC*^. The input *I*^*rec*^ consists of three components, *I*^*AMPA*^, *I*^*NMDA*^, and *I*^*GABA*^, representing the AMPA and the NMDA receptor-mediated excitatory currents, and the GABAA receptor-mediated inhibitory current, respectively. The currents *I*^*sc*^ and *I*^*sti*^ are mediated by AMPA receptor. The synaptic currents are given by:

(6) $$\begin{array}{l} I_{i}^{AMPA}\left(t\right)=g_{AMPA}\left(V_{i}-V_{E}\right)\overset{N_{E}}{\underset{j=1}{\sum }}W_{ij}C_{ij}S_{ij}^{AMPA}\left(t\right), \end{array}$$

(7) $$\begin{array}{l} I_{i}^{GABA}\left(t\right)=g_{GABA}\left(V_{i}-V_{I}\right)\overset{N}{\underset{j=N_{E}+1}{\sum }}W_{ij}C_{ij}S_{ij}^{GABA}\left(t\right), \end{array}$$

(8) $$\begin{array}{l} I_{i}^{NMDA}\left(t\right)=g_{NMDA}\left(V_{i}-V_{E}\right)\frac{\sum_{j=1}^{N_{E}}W_{ij}C_{ij}S_{ij}^{NMDA}\left(t\right)}{\left(1+C_{Mg}\exp\left(-0.062V_{i}/3.57\right)\right)}, \end{array}$$

where *g*_*AMPA*_, *g*_*GABA*_, *g*_*NMDA*_ denote the peak synaptic conductance of AMPA, GABA, and NMDA, respectively. *V*_*E*_ = *0mV* and *V*_*I*_ = −*70mV* are the excitatory and inhibitory reversal potentials, respectively. NMDA currents have a voltage dependence that is controlled by the extracellular magnesium concentration, *C*_*Mg*_ = 1 mM. *C*_*ij*_ = *1* if a connection exists from neuron *j* to *i*, otherwise *C*_*ij*_ = 0. *W*_*ij*_ is the synaptic weight from neuron *j* to *i* and is subject to STDP. $S_{ij}^{X}$(*X* = *AMPA, NMDA, GABA*) are synaptic gating variables (fraction of open channels). Following a spike arrival, the gating variables increase, or otherwise decay exponentially with time constants τ_*x*_.

The dynamics of the synaptic gating variables displaying STD are given by Tsodyks et al. (1998)

(9) $$\begin{array}{l} \frac{d{S_{ij}}^{AMPA}\left(t\right)}{dt}=-\frac{{S_{ij}}^{AMPA}}{\tau_{AMPA}}+Ux_{j}\left(t\right)\underset{k}{\sum }\delta \left(t-t_{j}^{k}-D_{ij}\right), \end{array}$$

(10) $$\begin{array}{l} \frac{dS_{ij}^{GABA}\left(t\right)}{dt}=-\frac{S_{ij}^{GABA}}{\tau_{GABA}}+\underset{k}{\sum }\delta \left(t-t_{j}^{k}-D_{ij}\right), \end{array}$$

(11) $$\begin{array}{l} \frac{dS_{ij}^{NMDA}\left(t\right)}{dt}=-\frac{S_{ij}^{NMDA}}{\tau_{NMDA,decay}}+y_{ij}^{NMDA}\left(t\right)\left(1-S_{ij}^{NMDA}\left(t\right)\right), \end{array}$$

(12) $$\begin{array}{l} \frac{dy_{ij}^{NMDA}\left(t\right)}{dt}=-\frac{y_{ij}^{NMDA}\left(t\right)}{\tau_{NMDA,rise}}+Ux_{j}\left(t\right)\underset{k}{\sum }\delta \left(t-t_{j}^{k}-D_{ij}\right), \end{array}$$

(13) $$\begin{array}{l} \frac{dx_{j}\left(t\right)}{dt}=\frac{1-x_{j}\left(t\right)}{\tau_{D}}-Ux_{j}\left(t\right)\underset{k}{\sum }\delta \left(t-t_{j}^{k}\right), \end{array}$$

where $t_{j}^{k}$ denotes the moment of the *k*th spike of neuron *j. D*_*ij*_ is the distance dependent transmission delay from neuron *i* to *j* (see the simulation protocol below for the detail definition). *U*∈(0, 1) denotes the utilization of the synaptic efficacy when a spike arrives. The variable y quantifies the efficiency of NMDA receptor at the post-synaptic neuron and the variable *x* quantifies the fraction of neurotransmitter available at the pre-synaptic neuron. τ_D_ is the depression time constant.

### The simulation protocol

We carry out numerical simulations to model the learning processes of the networks. The simulations were programmed in Python using Brian2 (Goodman and Brette, 2009) and NumPy libraries. Details of the model parameters are summarized in the Table 1.

**Table 1:** Model parameters.

**Network structure**		**Synapses parameters**	
One dimensional(1D) network size *N*	250	AMPA conductance *g_*AMPA*_*	1.2 nS
Number of excitatory neurons(1D) *N*_e_	200	NMDA conductance *g_*NMDA*_*	0.8 nS
Number of inhibitory neurons(1D) *N_*i*_*	50	GABA conductance *g_*GABA*_*	2 nS
Number of excitatory neurons(2D) *N_*e*×_ N_*e*_*	40 × *40*	NMDA receptor parameter *C_*Mg*_*	1
Number of inhibitory neurons(2D) *N_*i*×_ N_*i*_*	10 × *10*	AMPA synaptic time constant τ_*AMPA*_	2 ms
Distance between neurons Δ*x*	1	NMDA rise time constant τ_*NMDA,rise*_	2 ms
Simulation time step Δ*t*	0.1 ms	NMDA decay time constant τ_*NMDA,rise*_	200 ms
Connection width δ	10.8	GABA synaptic time constant τ_*GABA*_	5 ms
**Single neuron parameters**		STP time constant τ_*d*_	200 ms
Reset potential V_re_	−70 mV	STP parameter *U*	0.5
Firing threshold V_th_	−55 mV	Distance-dependent delay *D_*dist*_*	0.1 *ms*/Δ*x*
Exc. and Inh. reversal potential V_E_,V_I_	0 mV, −70 mV	**Input parameters**	
Leak reversal potential V_L_	−70 mV	Number of poisson spike noise *N_*bg*_*	100
Membrane capacitance C_m_	281 pF	Noise input conductance *g^*bg*^*	0.025 nS
Leak conductance g_L_	30 nS	Noise firing rate *r_*bg*_*	6 Hz
Slope factor Δ_*T*_	2 mV	Stimulus size *S_*sti*_*	5 Δ*x*
Adaptation time constant τ_*w*_	40 ms	Speed of moving stimulus *V_*sti*_*	3 Δ*x*/*ms*
Subthreshold adaptation *a*	4 nS	External conductance *g^*sti*^*	0.25 nS
Spike-triggered adaptation *b*	0.08 nA	Rate of external input *r_*sti*_*	200 Hz
Refractory time τ_*ref*_	2 ms		
**STDP parameters**			
Max.weight *W_*max*_*	1		
Positive learning rate *A_+_*	0.01		
Negative learning rate *A_−_*	0.0105		
Positive time constant τ_+_	20 ms		
Negative time constant τ_−_	20 ms		

#### 1D model

Excitatory neurons in LIP were distributed along a line were labeled from *1* to *N*_*E*_, while all inhibitory neurons were labeled from *1* to *N*_*I*_. The ratio between them was set to *4:1*. The distance between two excitatory neurons was normalized to *1*, hence the position of the *i*th excitatory neuron $x_{i}^{E}$ is *i/N*_*E*_. Inhibitory neurons were inserted into the chain at regular intervals, with $x_{j}^{I}$ denoting the position of the *j*th inhibitory neuron. Neurons *i* and *j* were connected with a probability $P_{ij}^{X,Y}=\exp\left(-\frac{{\left(x_{i}^{X}-x_{j}^{Y}\right)}^{2}}{2\delta^{2}}\right)$, where δ is a constant, and *X,Y* = *E,I* depending on the neuron type. The transmission delay from neuron *i* to *j* was given by $D_{ij}^{X,Y}=d|x_{i}^{X}-x_{j}^{Y}|$, with *d* as a constant. Before learning, the synaptic weight *W*_*ij*_*(t)* between LIP neurons was set to 0.1 for all connections.

The SC neurons modeled by a group of neurons firing according to Poisson process, which serve as the source of the CD signal. The duration of the CD signal depended on the magnitude of the saccade. The feedforward connection weight $W_{ij}^{SC}$(t) from SC neurons to LIP neurons was also set to a small value of 0.1 in the beginning.

All the neurons continually received background noises through the simulation, modeled by Gaussian white noises.

In the learning process, a moving stimulus $I_{i}^{sti}$(t) was injected into a group of nearby excitatory neurons (stimulus size *S*_*sti*_) for a fixed duration *S*_*sti*_*/v*_*sti*_ and slid from neuron *i* to neuron *N*_*E*_. Inhibitory neurons also received the moving stimulus when it passed by.

#### 2D model

The simulation protocol described above for the 1D model could be extended directly to the 2D case. We only considered 6 saccade directions for illustration. Every six neurons formed a cluster at a location in the 2D retinotopic map (Figure 2B). The 2D model was composed of *N*_*E*_× *N*_E×_ 6 excitatory neurons and *N*_*I*×_*N*_*I*×_6 inhibitory neurons. Denote $x_{I}^{E}$ and $y_{I}^{E}$ as the coordinate of the *i*th excitatory neuron in the 2D retinotopic map and $d_{I}^{E}$ as its preferred saccade direction. The inhibitory neurons were also inserted into the 2D retionatopic map at regular intervals. The coordinate of the *j*th inhibitory is $x_{j}^{I}$, $y_{j}^{I}$. Neurons *i* and *j* were connected with a probability,

(14) $$\begin{array}{l} P_{ij}^{X,Y}=\exp\left(-\frac{{\left(x_{i}^{X,Y}-x_{j}^{X,Y}\right)}^{2}+{\left(y_{i}^{X,Y}-y_{j}^{X,Y}\right)}^{2}}{2\delta^{2}}\right)\\ \exp\;\left(-\frac{{\left(d_{i}^{X,Y}-d_{j}^{X,Y}\right)}^{2}}{2{\delta_{d}}^{2}}\right) \end{array}$$

and transmission delay between neurons was given by

(15) $$\begin{array}{l} D_{ij}^{X,Y}=D\sqrt{{\left(x_{i}^{X,Y}-x_{j}^{X,Y}\right)}^{2}+{\left(y_{i}^{X,Y}-y_{j}^{X,Y}\right)}^{2}}, \end{array}$$

where *X,Y* = *E, I* depending on the neuron type. The initial values of weights were all set to *0.1*. Similarly, 6 groups of SC neurons sent Poisson spike trains to LIP neurons (Figure 2B), with each of them representing a saccade direction. Before learning, the couplings between SC to LIP were all-to-all without directional selectivity. The initial weights were also set to be *0.1*. Similar to the 1D model, in the learning process, a moving stimulus induced by a saccade swept over a group of LIP neurons.

## Results

### The learning mechanism

Before presenting the results, we first summarize the mechanism underlying the learning of the peri-saccadic RF remapping. At the developing stage of the brain, the network structure for remapping is not yet established. A saccade will cause a static visual image to sweep through the retina passively, creating the effect of the visual stimulus moving in the opposite direction to the saccade. During this process, LIP neurons located in the retinotopic map are activated sequentially by the moving stimulus; meanwhile, SC neurons holding the saccadic movement map respond, although the link between SC and LIP is not yet established. The visual experience described above is repeated many times. According to the STDP rule, a uni-directional connection path between LIP neurons is eventually learned; meanwhile, a matching connection from the saccadic direction in SC to the remapping direction in LIP is established. Over many such visual experiences in different retinotopic locations and different saccadic directions, a full 2D remapping CW model in LIP is finally developed.

### Learning to remap in the one-dimensional network

We first present the results after learning for the 1D network. As described in Materials and Methods, we apply an external input moving in the opposite direction of a saccade to the network, to reflect the passive shift of a static image along the retina caused by the eye movement. The CD signal is switched on from 100 ms before to 100 ms after the saccade. Synaptic plasticity occurs at the connections between excitatory neurons in LIP and the connections from SC neurons to LIP excitatory neurons.

**Figure 3:**
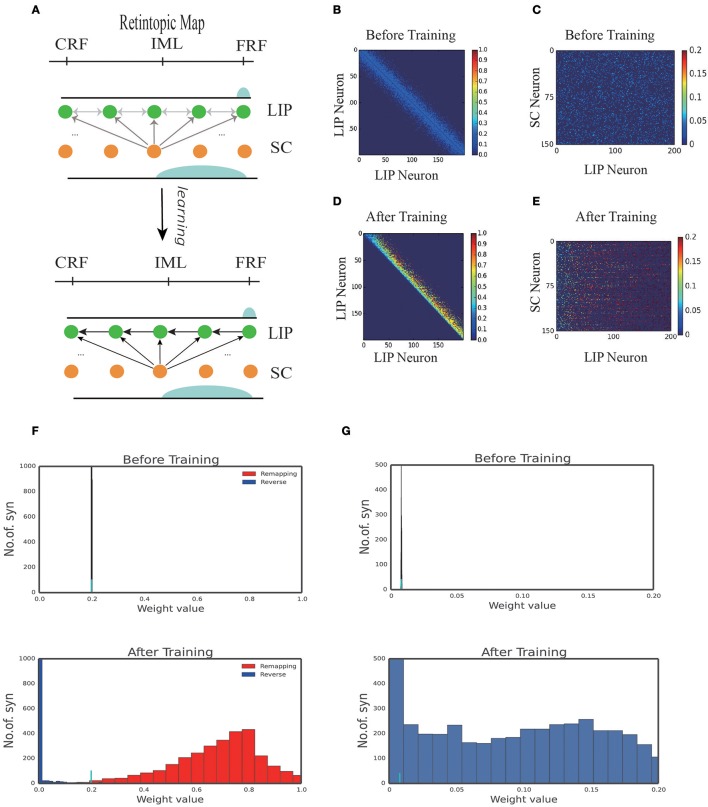
1D model results. **(A)** Illustration of the learning process. At the early stage of development, all the connections in the network are weak, hence no remapping occurs. After learning, a connection path in LIP in the opposite direction of the saccade is developed and the connection from SC to LIP is enhanced. **(B)** Connection pattern between LIP neurons before learning. **(C)** Connection pattern between LIP neurons and SC neurons before learning. **(D)** Connection pattern between LIP neurons after learning. **(E)** Connection pattern between LIP neurons and SC neurons after learning. **(F)** The distribution of connection weights between LIP neurons before and after learning. **(G)** The distribution of connection weights between LIP and SC neurons before and after learning.

Figure 3A displays the learned network structure after the network is exposed to the same moving input many times (experiencing saccades in the same direction for many times). Compared to Figure 2A, we see that enhanced are the neuronal synapses in LIP in the opposite direction of the saccade and the synapses from SC to LIP; weakened are the synapses in the direction of the saccade. Figures 3B,D displays changes in the weights of connections in LIP through learning. The initial weight matrix reveal no directional connectivity: the symmetry connections which are around the main diagonal imply bidirectional connectivity between neurons within the network. After learning with the directional moving stimulus, only the weights around the upper band of the main diagonal increase, which accounts for a potentiation of connections between neurons within the network in the direction of the moving stimulus. Figures 3C–E show that the connections from the SC neurons to the LIP neurons are also strengthened. Figures 3F,G present the weight distributions before and after learning.

**Figure 4:**
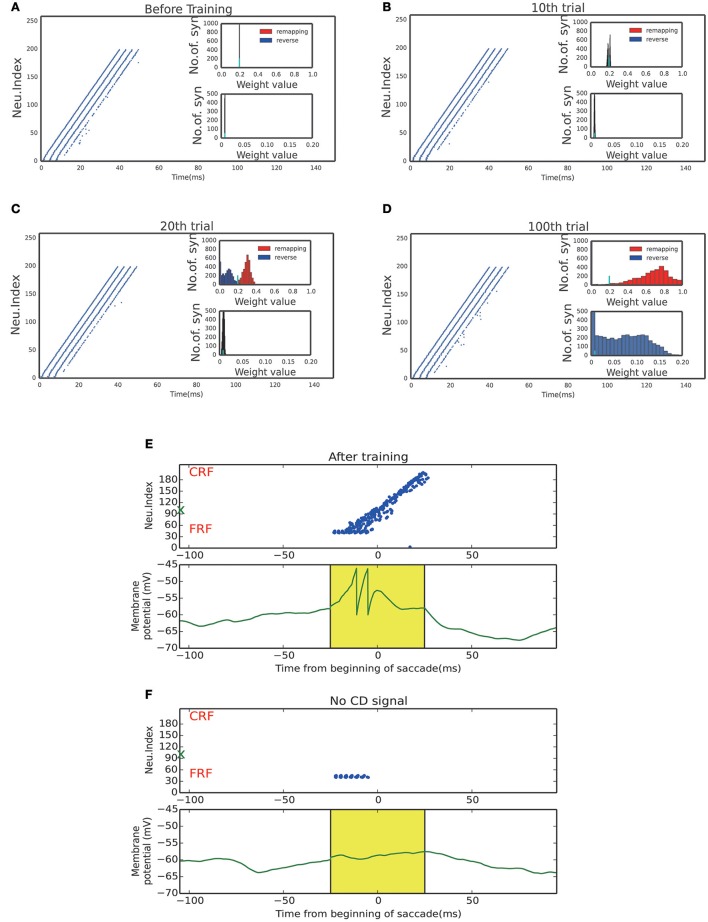
Activity raster and weight distribution of successful wave propagation before, during and after training. **(A–D)** snapshot of the training process, before learning, 10, 20, 100 trial, respectively. The insets illustrate the weight distribution changes along the training process. The remapping connections (red, in the opposite direction of saccade) are enhanced while the reverse connections (blue, in the direction of saccade) are weakened. The bottom insets show that the connections from the SC to LIP are strengthened by the training. The short cyan bars in the insets mark the initial value of the connection weights. **(E)** Upper: After training, a static stimulus at the start point causes a wave to propagate toward the end point. Bottom: membrane potential of the example neuron marked by green X in the upper panel. **(F)** In absence of the CD signal, no activity propagation is observed when loading a static stimulus onto the start point.

Figures 4A–D display the learning process of our model over trials. We see that as the time went on, the neuronal connections in LIP in the opposite direction of the saccade were gradually enhanced; whereas, the neuronal connections in the saccadic direction were gradually weakened. Furthermore, the connections from the SC to LIP were gradually strengthened during the learning.

The strengthened synapses at LIP form a unidirectional path, which works together with the timely CD signal to support the propagation of cortical wave. To test this, we apply a stimulus to a neuron at LIP simultaneously with the onset of the CD signal. As shown in Figure 4E, a cortical wave is initiated and propagates along the unidirectional path. We also see that without the CD signal, the stimulus alone is insufficient to activate a LIP neuron. The role of the CD signal is to increase the subthreshold membrane potential of a LIP neuron (Figure 4F), such that the neuron can be elicited by a visual stimulus or recurrent inputs from neighboring neurons.

**Figure 5:**
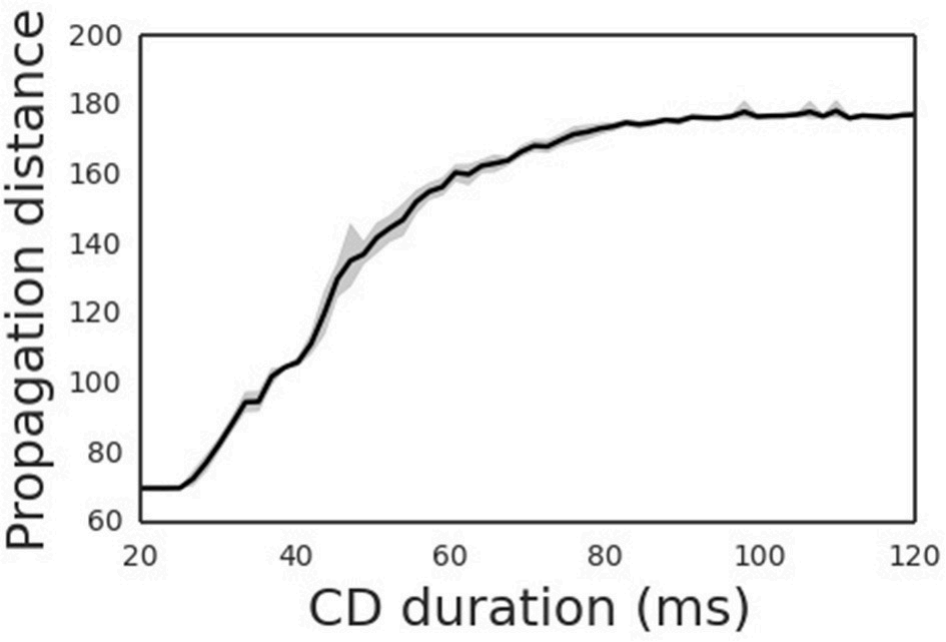
Propagation distances differ for different CD duration. The shade area is the 95% confidence interval of averaged propagation distance through CD duration.

The propagation distance of the cortical wave is determined by the duration of the CD signal (Figure 5), with the latter encoding the amplitude of the saccade. An experimental study revealed that the CD signal is associated with the moving hill activity in SC (Munoz and Wurtz, 1995). During the saccade, the moving hill activity migrates from the caudal to the rostral of the saccade map in SC. The saccade ends when the rostral colliculus begins to discharge. Thus, the duration of the CD signal correlates with the saccade amplitude. As expected, the propagation distance of the cortical wave monotonically increases with the duration CD signal. Thus, by correctly setting the duration of the CD signal, the cortical wave will propagate from the FRF to the CRF of a neuron, realizing remapping as required.

**Figure 6:**
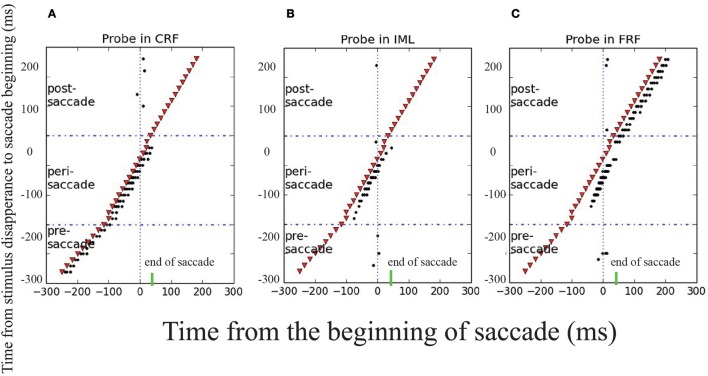
Transient elongation of visual receptive field around the time of a saccade. The y axes is the moments of applying the probe visual stimulus in different trials and the x axes is the time relative to the beginning of saccade. The saccade begins at *t* = 0 ms, represented by the solid and dashed vertical lines in each panel, the short green bars mark the end of saccade. A visual stimulus (red dot) is applied briefly at different moments (different rows) relative to the saccade in three locations: CRF **(A)**, IML **(B)**, and FRF **(C)**. The results show that: in the pre-saccadic period, the stimulus activates the neuron in CRF only; in the post-saccadic period, the stimulus activates the neuron in FRF only; but in the peri-saccadic period, the neuron is activated by the stimulus in all three locations, signifying the elongated remapping.

To reproduce the experimental findings as reported in Wang et al. (2016), we mimicked the experimental protocol and applied a probe stimulus to the CRF, IML, and FRF of a neuron at different moments with respect to the onset of the saccade. The results are shown in Figure 6, which strongly agrees with the experimental data, that is, (1) long before the saccade (100ms before), the neuron will only be activated by the probe stimulus at CRF; (2) about the time of the saccade, from 100 ms before and after, the neuron will be activated by the probe stimulus at CRF, IML, and FRF; (3) long after the saccade (after 100 ms), the neuron will only be activated by the probe stimulus at FRF. Overall, during the saccade, the RF of the neuron is effectively elongated along the saccade direction temporally. The underlying cause of this phenomenon is the propagation of cortical wave.

### Learning to remap in the two-dimensional network

We then present the results after learning for the 2D network. Since the movement of a stimulus is induced by a saccade, the direction a stimulus takes is determined by the direction of the saccade. In our model, each neuron in the column is assumed to respond selectively to a particular direction of a stimulus.

To mimic saccades with different saccade vectors, we chose a chain of neurons that “prefer” the same saccade direction in each training trial. We applied a stimulus moving in the opposite direction of the saccade to the chain. Therefore, chains of neurons embedded in the two-dimensional network are “sub-one-dimensional” (1D sub-network). Alike with the 1D model, the CD signal is turned on from 100 ms before to 100 ms after the saccade and synaptic plasticity occurs at the connections between excitatory neurons in LIP and the connections from SC neurons to LIP excitatory neurons.

**Figure 7:**
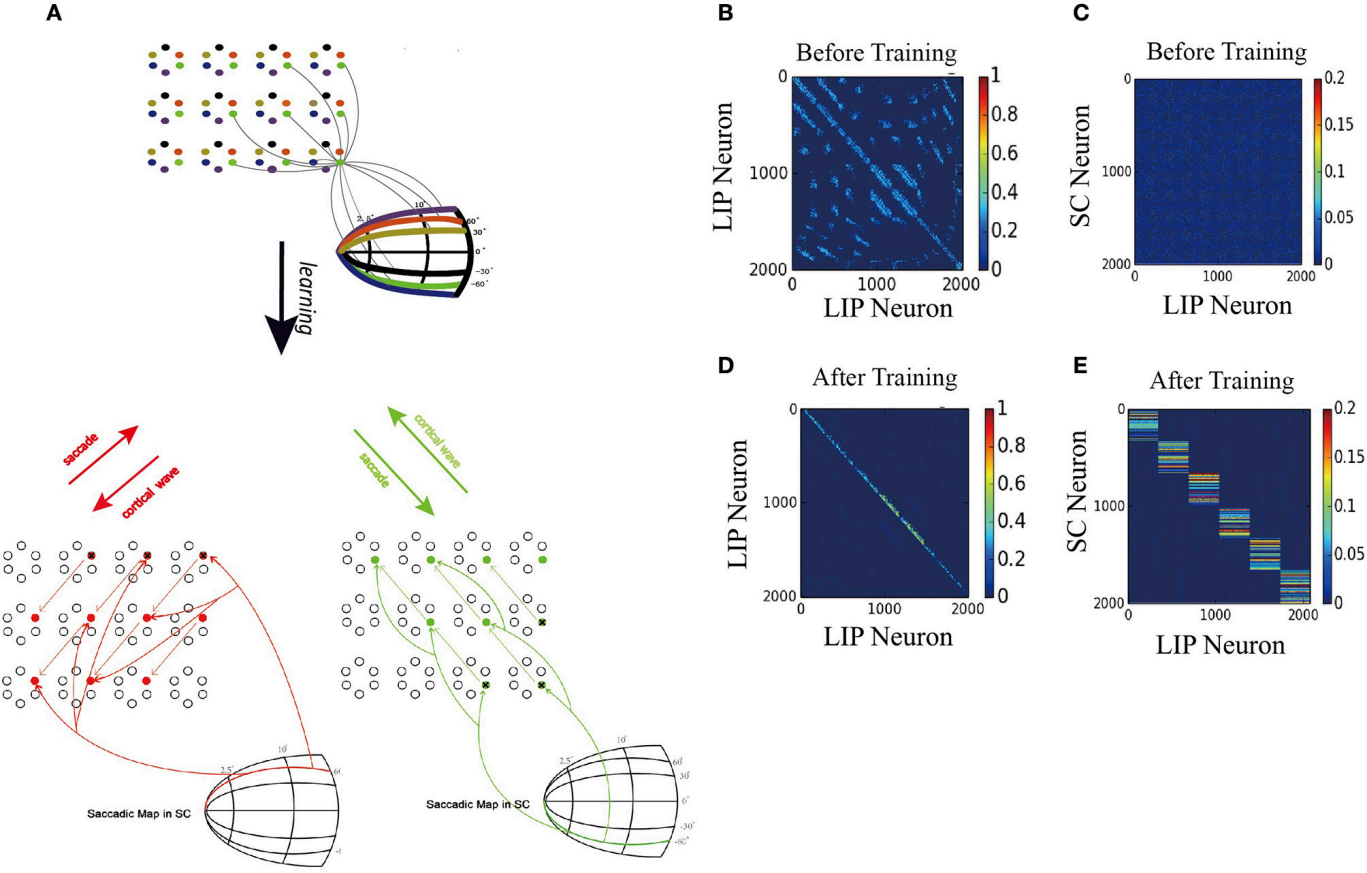
2D model results. **(A)** Schematic of the two-dimension model. Upper: The network is composed of many mini-columns are arranged on a 2D grid. Each mini-column is composed of 6 pyramidal cells which preferentially active for one direction of moving stimulus. Bottom left: network is trained by 60° upward saccades. The red arrows in LIP indicate the strengthening of uni-directional connections between neurons preferring that direction. The red arrows from saccade map in SC to the LIP neurons show that after training, coupling between SC and LIP neurons reveals direction selectivity. Bottom right: 60° downward saccades, similar with 60° upward saccades. **(B)** Initial connectivity matrix of LIP, Neurons are sorted according to their preferred directions and which sub-network they belong to. **(C)** Initial connectivity matrix of SC to LIP. **(D)** Matrix of synaptic weights of LIP, after training. Uni-directional connections have been built up while other connections are suppressed. **(E)** Matrix of synaptic weights of SC to LIP, after training. Connections between SC neurons and LIP neurons that code the same saccade direction are enhanced, otherwise suppressed.

Figure 7A displays the learned network structure after the network is exposed to a stimulus moving in different directions (experiencing eye movements with different saccade vectors for many times). Compared to Figure 2B, the 2D network has been modified by the STDP learning rule as follows. (1) Repeated experiences of saccades in one direction cause a chain of neurons to select a sub-1D network. The uni-directional connections which are opposite to the saccade directions are strengthened, while the connections in the direction of the saccade are weakened. (2) Synapses between the neurons in other sub-1D networks are pruned. (3) A saccade map in SC that provides information about saccade direction and amplitude allows strengthened coupling between the “selected” LIP neurons (1D sub-network) and the SC neurons in the same saccade map, while weakening coupling between the other “non-selected” LIP neurons(1D sub-network preferring other saccade directions) and the chosen group of SC neurons. These modifications of the network ensure that the propagation of the cortical wave is constrained to a one-dimensional path from the FRF to CRF.

To support the propagation of cortical wave, the strengthened synapses at LIP work together with the timely and directional CD signal, similar to the 1D model. Specifically, when the monkey is looking at FPa (Fixation Point) and planning a 60° right upward saccade, all neurons preferring that direction will receive a set of CD inputs, which increase the subthreshold membrane potential of neurons in the 1D sub-network (see the red arrow stemming from the 60° curve in the saccade map of SC Figure 7A). When the monkey is looking at FPb and planning a 60° right downward saccade, the sub-1D network with neurons that prefer this direction will receive the CD signal that codes the 60° right downward saccade (see the green arrow from the −60° curve in the saccade map of SC Figure 7A).

Figures 7B,D depict changes in the weights of connections in LIP and the emergence of direction-specific connectivity through learning. Neurons are grouped according to their preferred direction of saccade and the sub-network they belong to. The initial weight matrix reveal no directional connectivity: (1) the symmetry connections which are around the main diagonal imply bidirectional connectivity between neurons within a sub-network; (2) other connection patterns represent the connections between neurons in different sub-network. After learning with the directional moving stimulus, only the weights around the upper band of the main diagonal increase, which accounts for a potentiation of connections between neurons within the same sub-network in the direction of the moving stimulus. Other connections are weakened and pruned in the ongoing training process.

Furthermore, the connections from the targeted SC neurons to the LIP neurons that share the same saccade direction preference are strengthened, otherwise, weakened (Figures 7C,E). This connection pattern can support the propagation of the cortical wave by conveying the CD signal properly to LIP neurons according to the saccade vector.

**Figure 8:**
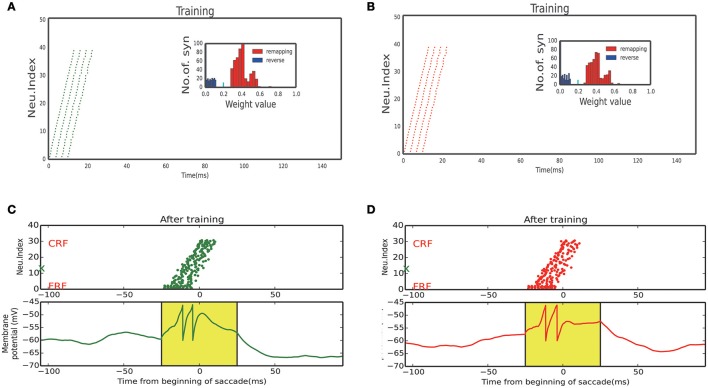
Neuron response of sub-network. **(A)** Activity raster and weight distribution when training with 60° upward saccades (Figure 7A, Bottom left). **(B)** Activity raster and weight distribution when training with 60° downward saccades (Figure 7A, Bottom right). **(C)** A static stimulus (Black marker in Figure 7A, when monkey plan a 60° upward saccade) at FRF, a wave propagates toward CRF. Bottom: membrane potential of the example neuron marked by red X in the upper panel. **(D)** A static stimulus (Black marker in Figure 7A, when monkey plan a 60° downward saccade) at FRF, a wave propagates toward the CRF. Bottom: membrane potential of the example neuron marked by green X in the upper panel.

After training, the strengthened synapses at LIP form many unidirectional paths. Together with the timely CD signal, they support the propagation of the cortical wave in the 2D network. As shown in Figure 7A, when a static visual stimulus triggers the neuron with a FRF, a cortical wave is initiated and propagates along the 1D path corresponding to the saccade vector. The responses of neurons on the path are illustrated in Figures 8C,D, 60° upward saccade and 60° downward saccade, respectively.

We stimulated the plastic network with a stimulus moving in different directions according to a random sequence to reflect a visual experience involving different saccades. Neurons that received the moving stimulus fired sequentially (Figures 8A,B, response of 60° and −60° sub-network, respectively), resulting in the connections which in the same direction with the moving stimulus exhibit a positive development while a negative development in the opposite directions (inset of Figures 8A,B).

## Conclusion

In this study, we have built a spiking neural network model with synaptic plasticity to demonstrate that the peri-saccadic remapping function can be acquired from visual experiences at the development stage of brain. Our model implemented the main results of the previous modeling work and reproduced a number of experimental results. We showed that, with the learning process, the unidirectional connections between the LIP neurons were strengthened as well as the couplings between LIP and SC. When the network was trained, the CD signal was able to facilitate a transcortical spread of activity which was triggered by a static visual stimulus at FRF of a neuron, and this activity would propagate to CRF of the neuron, resulting in the remapping phenomenon. To display the receptive field elongation, we also showed that a probe stimulus at the IML would drive the neuron with CRF around the time of saccade. We also expanded the 1D model to implement the saccades in a 2D space, and showed that direction-specific connections emerged in the learning process. Specifically, a visual stimulus in 2D space guided the learning process to strengthen the connectivity between LIP neurons with the same preference of direction, while the connections from SC neurons to LIP targeted neurons that prefer the same saccade direction were also strengthened. We hope that this study strengthens our understanding on the function of RF remapping.

## Author contributions

MZ and SW designed the project. XW carried out the simulations and made the figures. XW, YW, and SW wrote the manuscript.

### Conflict of interest statement

The authors declare that the research was conducted in the absence of any commercial or financial relationships that could be construed as a potential conflict of interest.
